# Myeloid sarcoma: an uncommon presentation of myeloid neoplasms; a case series of 4 rare cases reported in a tertiary care institute

**DOI:** 10.4322/acr.2021.339

**Published:** 2021-11-05

**Authors:** Toyaja Jadhav, Puneet Baveja, Arijit Sen

**Affiliations:** 1 Armed Forces Medical College, Department of Pathology, Pune, Maharashtra, India

**Keywords:** Sarcoma, Myeloid, Leukemia, Myeloid, Acute, Leukemia, Promyelocytic, Acute, Leukemia, Myelogenous, Chronic, BCR-ABL Positive, Myelodysplastic Syndromes

## Abstract

Myeloid sarcoma (MS) is a rare extramedullary neoplasm of myeloid cells, which can arise before, concurrently with, or following hematolymphoid malignancies. We report 04 such cases of MS, diagnosed in this institute over a period of 6 years, during various phases of their respective myeloid neoplasms/leukemias. These cases include MS occurring as a relapse of AML (Case 1), MS occurring as an initial presentation of CML (Case 2), MS occurring during ongoing chemotherapy in APML (Case 3), and MS presenting as a progression of MDS to AML (Case 4). In the absence of relevant clinical history and unemployment of appropriate immunohistochemical (IHC) studies, these cases have a high risk of being frequently misdiagnosed either as Non-Hodgkin’s Lymphoma (NHL) or small round cell tumors or undifferentiated carcinomas, which may further delay their management, making an already bad prognosis worse. This case series has been designed to throw light on the varied presentation of MS and the lineage differentiation of its neoplastic cells through the application of relevant IHC markers along with their clinical correlation.

## INTRODUCTION

Myeloid sarcoma (MS), also known as Granulocytic Sarcoma or Chloroma, is a rare extramedullary manifestation of hematological malignancies of myeloid origin. It is characterized by the formation of clinically evident tumors containing immature myeloid cells in extramedullary sites, commonly involving the skin, soft tissues, central nervous system (CNS), and the urogenital tract.

Here, we report a series of 04 cases of MS reported in this institute over 06 years, diagnosed histopathologically during different clinical phases of their respective myelogenous neoplasms/leukemias.

## CASE REPORTS

### Case 1

A 13-year-old female presented to a tertiary care hospital with a painless lump in her right breast in the lower outer quadrant, noticed by her 02 days ago. Her past medical history revealed Acute Myeloid Leukemia (AML) with cytogenetic abnormality t(8;21), detected 9 months before. Flowcytometry had revealed AML with the expression of CD1 and CD56. She was managed with AML BFM protocol for 7 months followed by cranial irradiation, after which she was followed up with bone marrow and cerebrospinal fluid (CSF) examination, which connoted remission.

Fine needle aspiration cytology (FNAC) examination of the lump was done for preliminary assessment, where largely, the features suggested a hematolymphoid neoplasm. Histopathological evaluation (HPE) was indicated in this case for a definitive diagnosis of an extramedullary hematolymphoid neoplasm in the breast lump.

**Figure 1:**
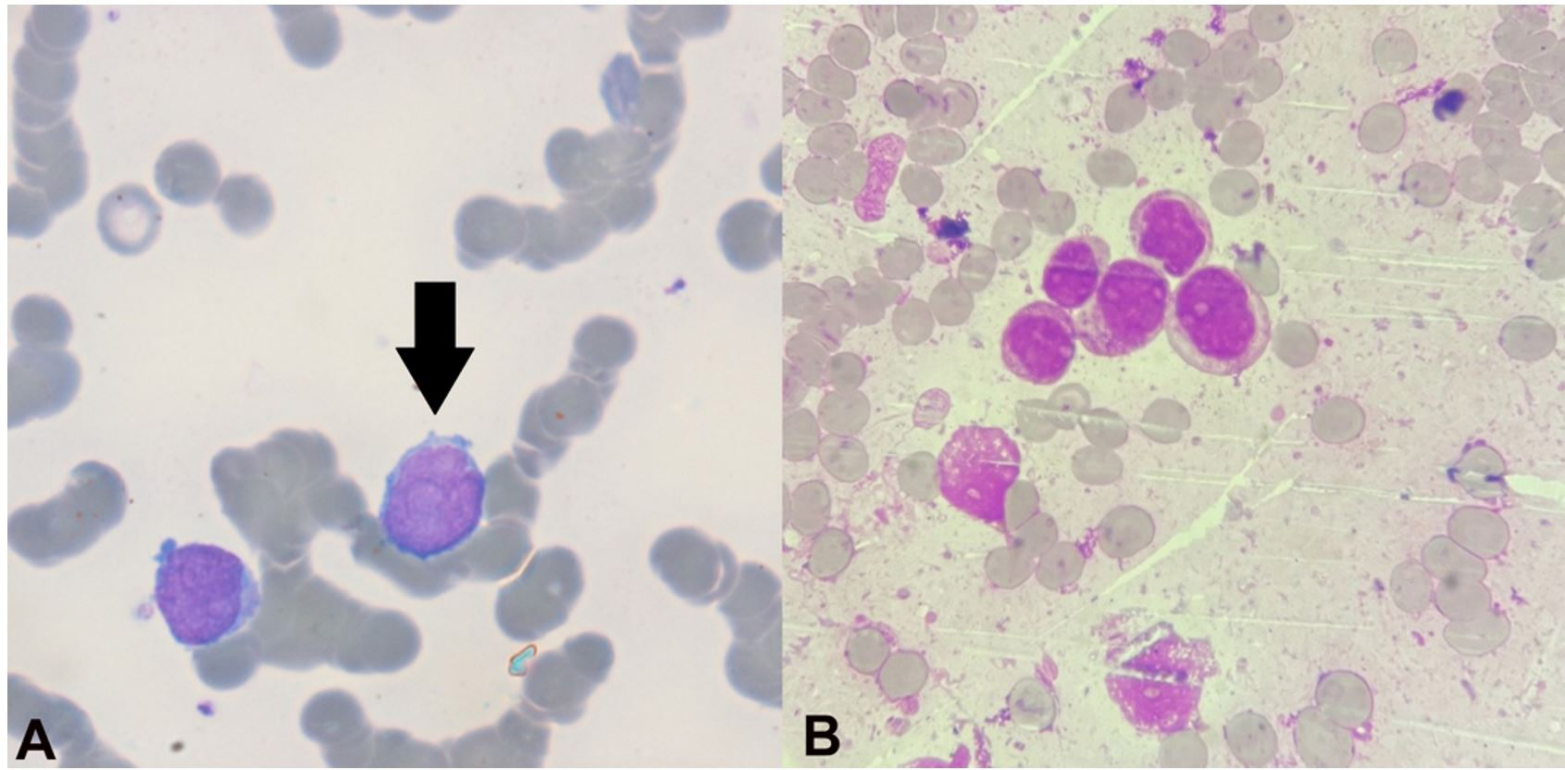
**A** – Peripheral blood smear showing blasts (arrow). (Leishman's stain, 1000x); **B** – Bone marrow aspirate smear showing blasts of myeloid lineage (Leishman's stain,1000x)

Concurrently, this patient also underwent a complete hematological examination. Peripheral blood (PBS) showed 8% blasts. Bone marrow evaluation displayed 61% blasts of myeloid lineage (Figure 1), which accorded the relapse of AML. Bone marrow karyotyping was also done, which showed an additional trisomy of chromosome 15, in a known case of AML with t(8;21).

On excision, the breast lump was sent for histopathological assessment.

**Figure 2:**
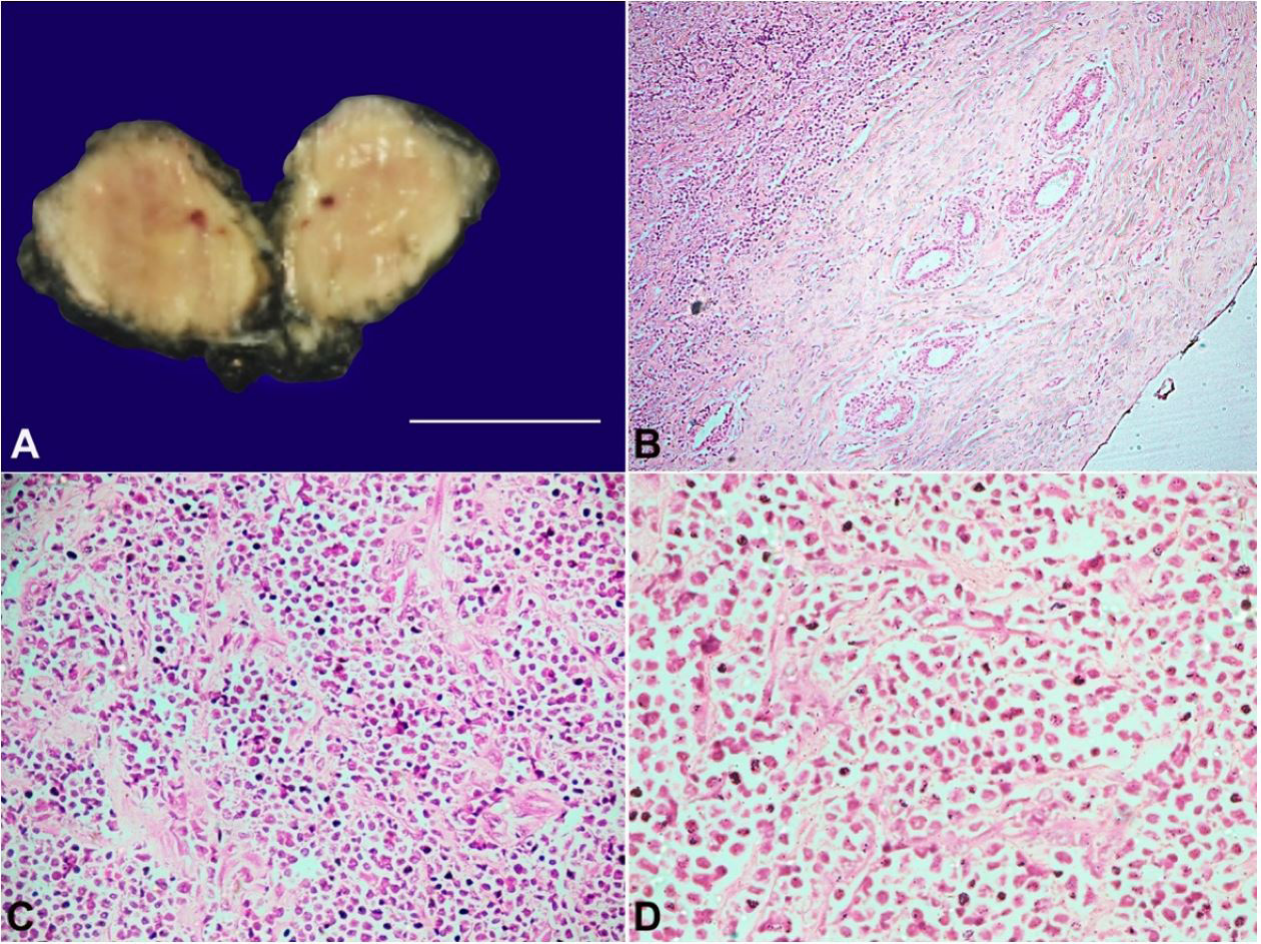
**A** – A gross view of the excised breast lump. Note the external surface appears smooth with the cut surface showing a greenish tinge (scale bar = 4 cm); **B, C** and **D** show sections from the breast mass, which reveal tumor cells composed of monomorphic population of cells, predominantly composed of blasts along with few scattered mature and immature granulocytes. Normal breast parenchyma is seen at the periphery in B, indicating that the tumor was circumscribed (H&E, B-100x; C - 200x; D - 400X).

Grossly, the lump appeared as a fibroadipous tissue mass measuring 4×3×1.5cm. The external and cut surface was smooth and yellowish white and had a greenish tinge. No lobulations or raw areas were noted on the surface, neither were necrotic or hemorrhagic foci (Figure 2A).

The microscopy of the lump revealed a well-circumscribed, non-encapsulated neoplasm constituting a monomorphic population of cells, mainly composed of blasts along with a few mature and immature granulocytes. These blasts had moderate to minimal cytoplasm with occasional granularity. A high nuclear-cytoplasmic (N:C) ratio was seen, with oval to round nuclei showing marked anisonucleosis and vesicular chromatin. Inconspicuous nucleoli were present in some cells. Few mitotic figures were noted, with the presence of atypical mitoses. The periphery of the tumor showed sparse normal breast parenchyma with terminal ductal lobular units (TDLUs) (Figure 22C, and 2D). The surgical resection margins were free from the tumor.

**Figure 3:**
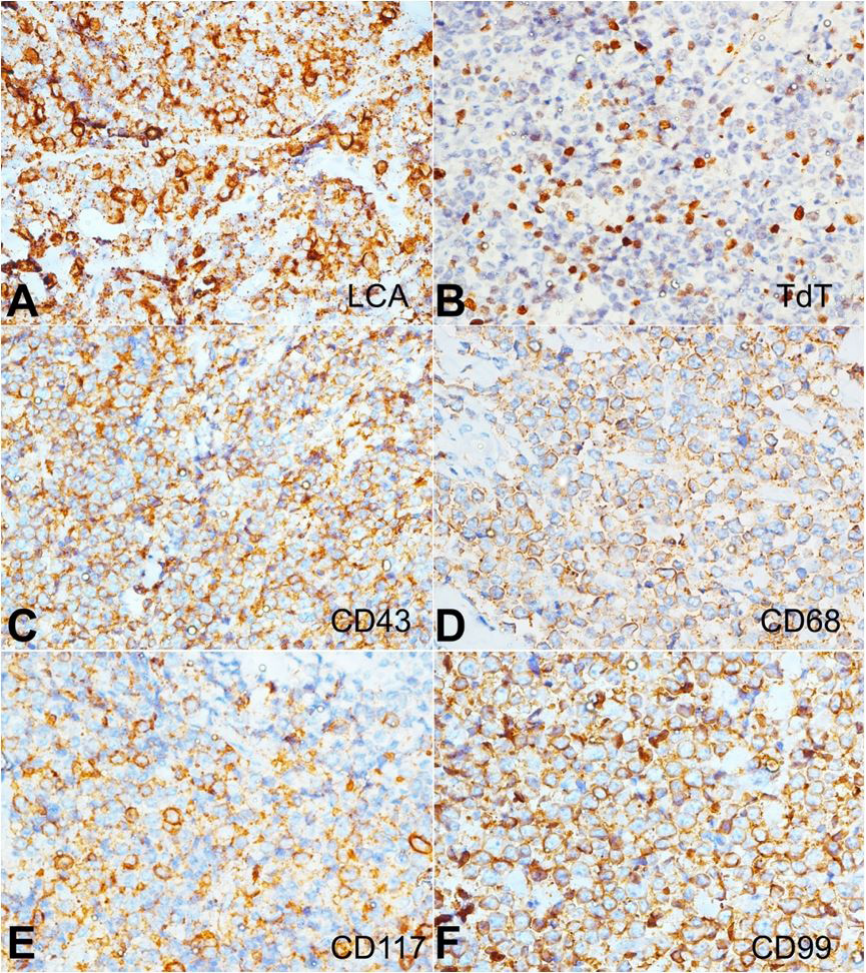
- Immunohistochemical profile of the breast lump - The tumor cells are strongly reactive for **A** – LCA; **B** – TdT highlights the myeloblasts; **C** – The tumor cells also react diffusely to CD43; **D** – CD68 (diffuse reactivity); **E** – CD117 (intermediate to weak reactivity); and **F** – CD99 (diffuse reactivity) (A to F, 400X).

On immunohistochemical analysis using anti-myeloperoxidase (MPO) antibodies, the tumor cells showed strong cytoplasmic reactivity. On further immunohistochemical profiling, the tumor cells showed strong immunoreactivity to antibodies against CD43, CD56, CD68, Leucocyte common antigen (LCA), and CD99 with an intermediate to weak reaction to CD117 and TdT (Figure 3), while they were non-reactive for lymphoid B and T cell markers.

These microscopic features, along with immunohistochemical studies, established the diagnosis of myeloid sarcoma. The same, along with hematological features, was considered as relapse of AML, which was treated accordingly.^1^ The patient succumbed to the disease 04 months after diagnosis of MS.

### Case 2

A 70-year-old male, with no previous history of any hematological abnormality, presented with a swelling in the right side of the neck for 03 months. On examination, it was an enlarged right cervical lymph node. A lymph node excision biopsy was conducted with simultaneous hematological evaluation.

**Figure 4:**
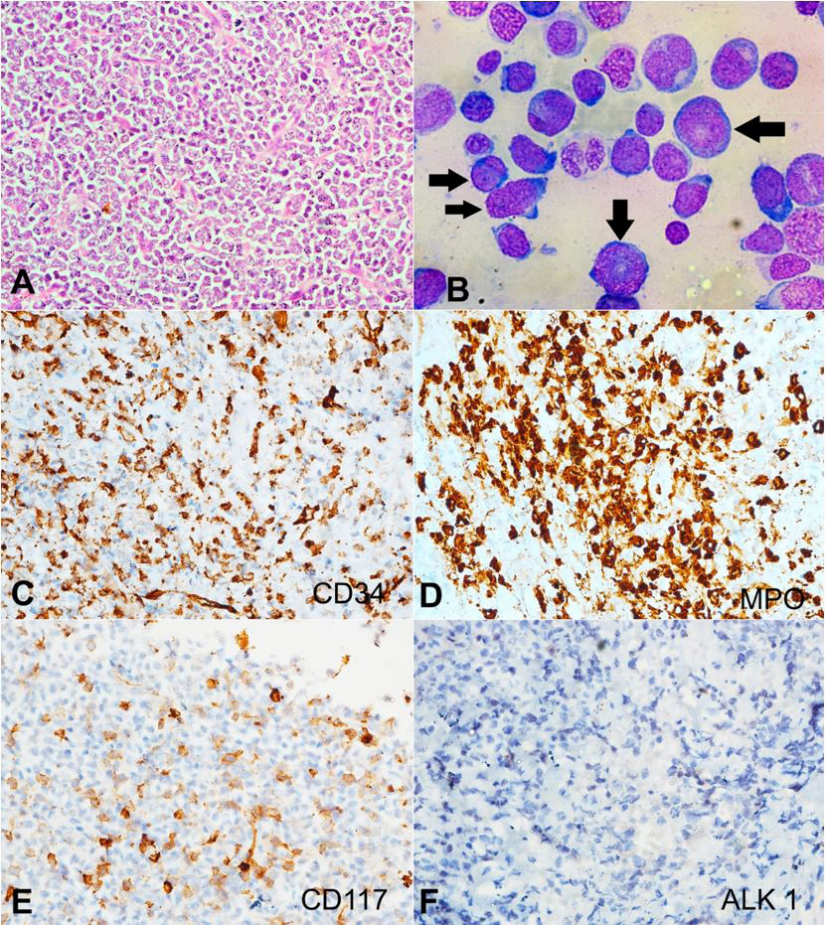
**A** – Photomicrograph of the lymph node showing the replacement of lymph node parenchyma with sheets of blasts (H&E, 400x); **B** – Bone marrow aspirate showing numerous blasts (arrows) along with other cells of myeloid lineage, indicating blast crises of CML (Leishman’s stain,1000x); **C-F** – Immunohistochemical profile of the lymph node reveals the blasts being diffusely positive for: C - CD34; D – MPO; E - focal reactivity to CD117; and F - non-reactivity to ALK1 - (C to F, 400x).

Microscopic evaluation of the excised lymph node revealed fragmented tissue bits consisting of lymphoid tissue with a distorted and effaced architecture showing replacement of the entire parenchyma by intermediate-sized proliferating lymphoid cells. These cells had a high N:C ratio with a small rim of cytoplasm, vesicular nuclei, and a stippled chromatin with prominent nucleoli. Numerous centrocyte-like cells having cleaved nuclei were also noted. Scattered mitosis was noted with focal hotspots showing atypical mitosis and 1-2 mitotic figures/High power field (HPF). Scattered plasma cells were noted in a few foci. No tingible body macrophages or necrosis was noted. Based upon these attributes, besides the relevant immunohistochemistry showing focal positivity for CD3 and CD5, an initial diagnosis of Non-Hodgkin’s Lymphoma (NHL) favoring T cell lymphoblastic leukemia was entertained (Figure 4A).

The hematological evaluation revealed a PBS consisting of neutrophilic leukocytosis with 15% blasts and no basophilia. However, bone marrow studies revealed 70% myeloid blasts (Figure 4B), along with t(9;22) on cytogenetic evaluation, which was consonant with a diagnosis of CML – Blast crisis.

Hence, the lymph node biopsy was reviewed with concurrent PBS and bone marrow findings. On additional immunohistochemical evaluation, the atypical cells within the lymph node, now established as blasts, showed reactivity for CD34 along with MPO, CD43, CD56, and CD117 with a Mib-1 labeling index of 70-80% (Figure 4C-F). These features confirmed the diagnosis of Myeloid sarcoma in a freshly detected case of CML with blast crises.

The patient was initially managed with Imatinib and hydroxyurea combination, to which he initially responded. However, he progressed to blast crises again after a year, following which he was administered Ponatinib + FLAG-Ida therapy. The patient, however, succumbed to his illness within 02 months of relapse.

### Case 3

A 5-year-old male child presented with swelling in the left temporal region over the last 15 days. On evaluation, he was known to be a newly diagnosed case of acute promyelocytic leukemia (APML) with the absence of t(15;17), but having del(16q), 02 weeks before noticing the presence of the head swelling. He was receiving chemotherapy in the form of All-trans retinoic acid (ATRA) since the diagnosis.

FNAC examination of the swelling was done for preliminary assessment along with concurrent hematological workup.

**Figure 5:**
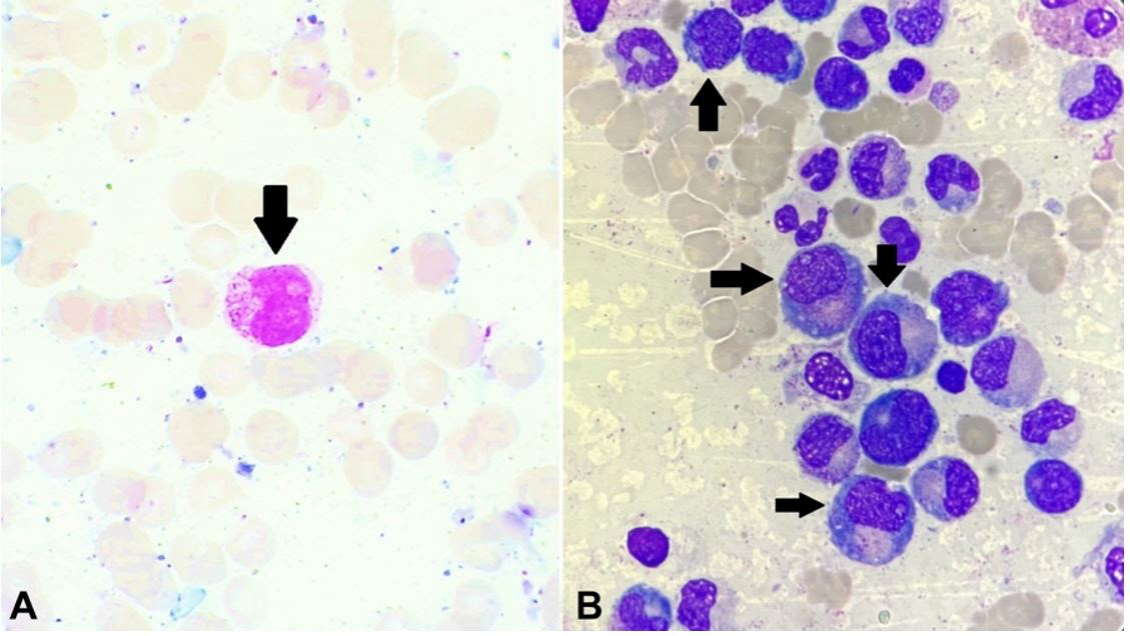
**A –** Peripheral blood smear showing an abnormal promyelocyte composed of an eccentric folded nucleus with nucleolus and abundant cytoplasm showing numerous granules along with the presence of Auer rods (arrow) (Leishman's stain, 1000x); **B** – Bone marrow aspirate shows promyelocytes (arrows) along with a few blasts and myeloid precursors (Leishman's stain, 1000x)

PBS showed 33% blasts with promyelocytes. Bone marrow assessment revealed 28% promyelocytes of hypergranular type (Figure 5A and 5B), which was compatible with a diagnosis of APML.

Flowcytometric analysis revealed Acute myeloid leukemia with the absence of CD34 and HLA-DR.

**Figure 6:**
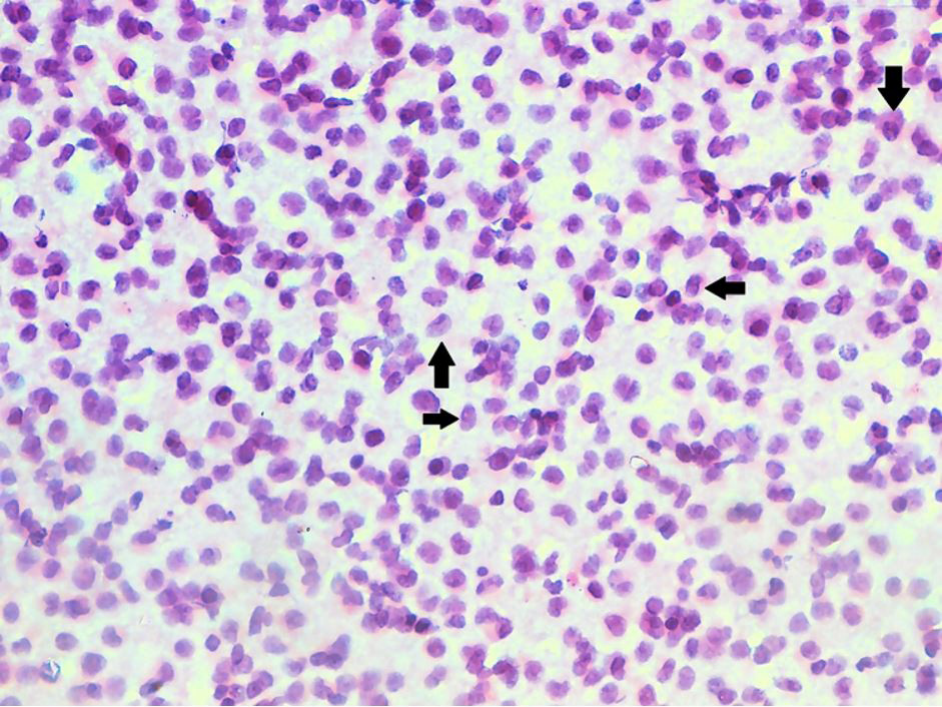
May Grunwald - Giemsa (MGG) stained smears from the FNAC of temporal swelling reveal a monomorphous population of cells composed of intermediate-sized cells with pinkish granular cytoplasm and prominent nucleoli (arrows), suggesting overall features to be of a hematolymphoid malignancy in a known case of APML (400x).

The aspirate smears from the left temporal region were cellular and revealed monotonous intermediate-sized atypical cells with moderate cytoplasm and eccentrically placed nuclei having open chromatin and some showing prominent nucleoli. Many cells also showed reniform nuclei suggesting overall features to be of a hematolymphoid neoplasm in a known case of APML (Figure 6).

He was also evaluated radiologically. The contrast-enhanced CT scan (CECT) revealed an extensive periosteal reaction involving bones of the skull base and mandible with overlying enhancing soft tissue swelling extending into the extradural space, favoring a granulomatous etiology with the differential diagnosis of hypervitaminosis A, late-onset Caffey’s disease and infiltration by leukemia/lymphoma or a small round blue cell tumor.

He also underwent MRI brain + orbit, the findings of which revealed enhancing soft tissue masses within the maxillary and sphenoid sinuses with bony involvement showing hair on end type of periosteal reaction. These findings were suggestive of a possible extramedullary hematopoiesis with a differential diagnosis of a myeloproliferative disorder.

HPE was indicated in this patient for further assessment of the lesion and revealed the presence of numerous atypical cells showing a high N:C ratio, with most of them having convoluted nuclei. These atypical cells showed reactivity for MPO and CD117 immunohistochemically, confirming their myeloid lineage. Hence, these features affirmed the diagnosis of myeloid sarcoma in a known case of APML undergoing therapy. There was no change in the management protocol and the patient continued to receive ATRA therapy. However, he succumbed to the illness within a month of diagnosis of MS.

### Case 4

A 49-year-old female presented with vaginal bleeding and anemia. The gynecological assessment revealed a uterocervical mass. On evaluation, there was a history of myelodysplastic syndrome - excess blast variant (MDS EB2) with monosomy 7 (confirmed on FISH studies), diagnosed a year ago. She was managed by Decitabine therapy, after which she was followed up with bone marrow studies, which demonstrated remission.

She underwent total hysterectomy with bilateral salpingo – oophorectomy (TAH+BSO) for the uterocervical mass.

**Figure 7:**
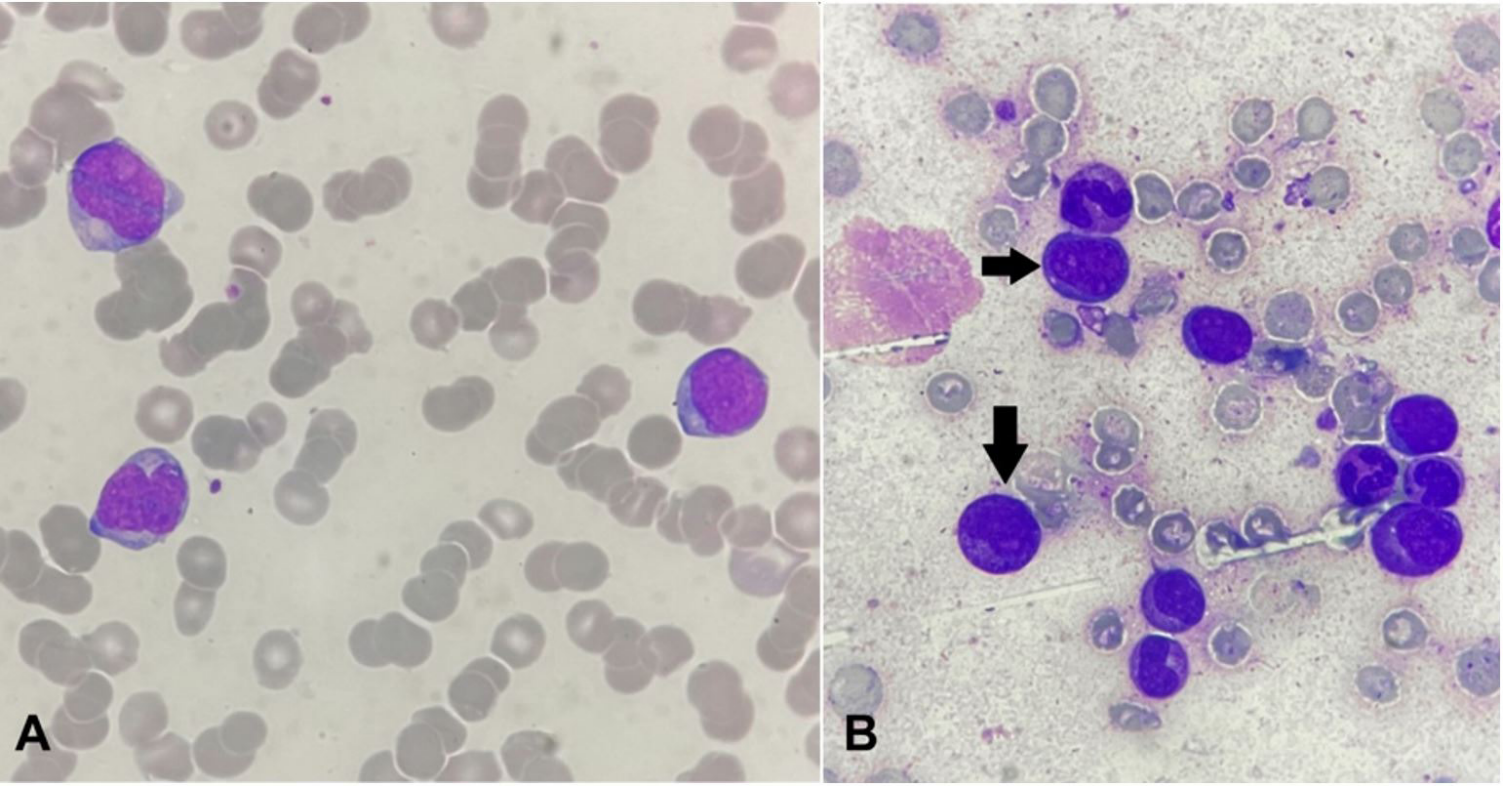
**A** – Peripheral blood smear showing the presence of myeloid blasts (Leishman stain,1000x); **B** – Bone marrow aspirate reveals presence blasts (arrows) with cells of myeloid lineage (Leishman stain, 1000x).

Concurrent PBS showed 15% blasts (Figure 7A). Flowcytometry revealed positivity for CD4, CD11c, CD33, CD38, CD45, CD34, HLA-DR, CD117, Cd11b, CD13, CD14 and MPO. Bone marrow assessment revealed 24% blasts along with promyelocytes (Figure 7B), which established the diagnosis of secondary AML following the transformation of MDS - EB2.

**Figure 8:**
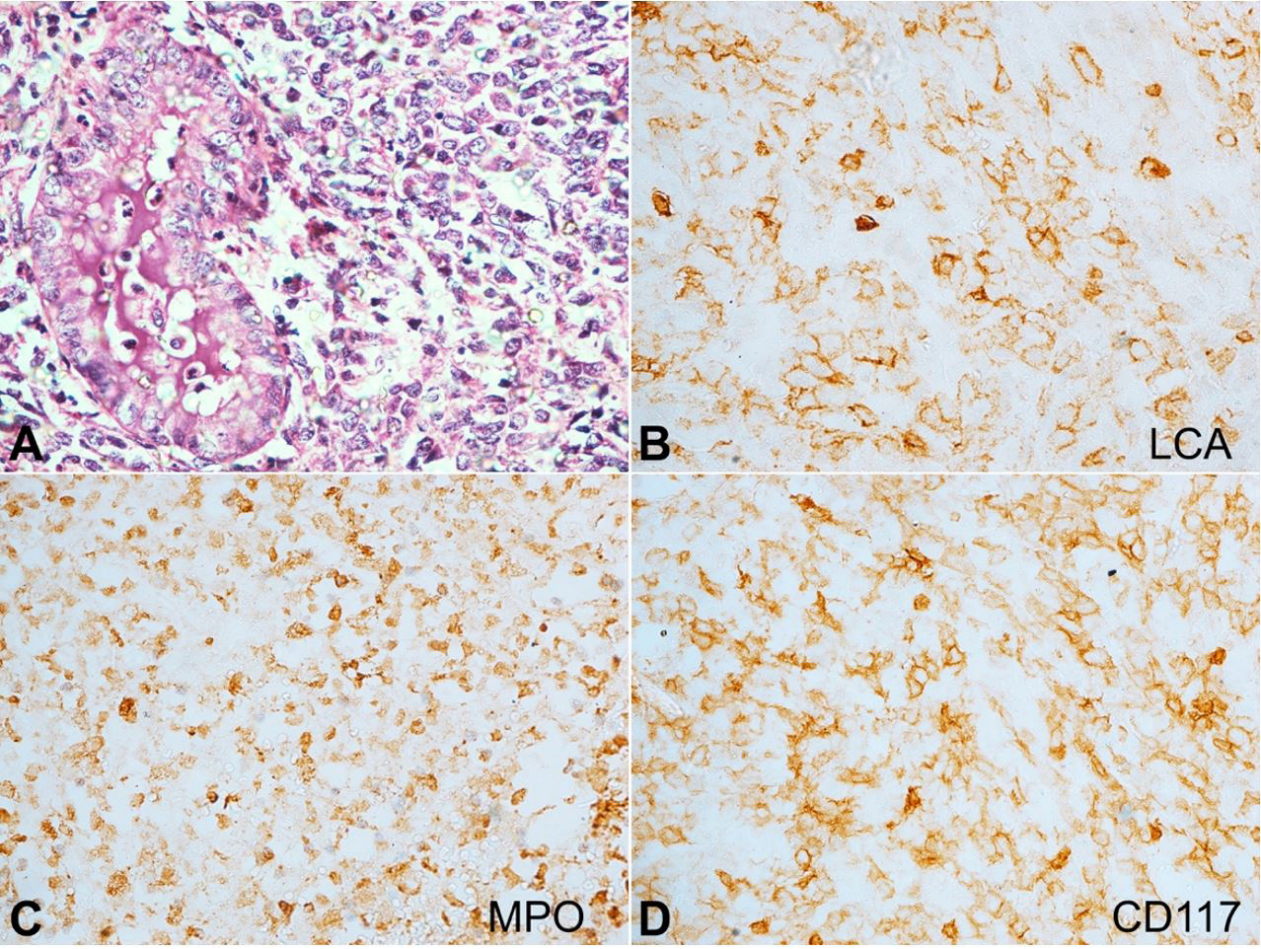
Photomicrographs of the cervical mass: **A** – shows scant endocervical glands along with stroma being infiltrated by atypical monomorphic cells in sheets (H&E, 400x); **B-D** – Immunohistochemical reactions reveal the atypical cells showing strong reactivity to: B - LCA, C – MPO, and D – CD117, indicating their myeloid lineage (400x).

Microscopic evaluation of the cervical mass revealed focal endocervical lining and few endocervical glands with stroma showing infiltration by atypical monomorphic cells arranged in sheets, cords, and singly scattered, at places encroaching upon the endocervical glands (Figure 8A). These atypical cells were large to medium in size and exhibited a high N:C ratio, scant cytoplasm, round to oval nucleus, open chromatin and few prominent nucleoli. Areas of necrosis were seen along with occasional atypical mitotic figures. The endometrium, myometrium and adnexa were uninvolved. On immunohistochemical evaluation, the atypical cells showed reactivity for MPO, LCA, and CD117 (Figure 8B-D). These microscopic characteristics, along with immunohistochemical profile, confirmed the diagnosis of myeloid sarcoma in a case of secondary AML, indicating the leukemic transformation of MDS - EB2.

She was again started on Decitabine therapy, as her kin refused salvage chemotherapy. However, she succumbed to the illness after 09 months of diagnosis of MS.

Table 1 and 2 summarize the cases.

**Table 1:** Histomorphological and immunohistochemical features

case	Microscopy	CD34	MPO	CD117	CD43	CD56	Diagnosis
1	Unencapsulated tumor, Monomorphic population of cells, Blasts and mature and immature granulocytes	+	+	+	+	+	MS
2	Effacement of nodal architecture, Sheets of blasts, Numerous atypical mitosis	+	+	+	+	+	MS
3	Tumor composed of monomorphic intermediate sized cells, Cells show nuclear grooving, Numerous buttock cells seen	-	+	+	NA	-	MS
4	Focal endocervical glands, Stroma infiltrated by monomorphic cells in sheets and singly scattered., Atypical cells are blasts	NA	+	+	NA	NA	MS

NA = non-available; MS = myeloid sarcoma.

**Table 2:** Blood and bone marrow findings

Case	Blood picture	Bone marrow	Cytogenetic Studies	Treatment administered	Pt outcome
1	8% blasts	61% blasts (AML relapse)	t(8;21)	FLAG-Ida regime of salvage chemotherapy	Death after 04 months of diagnosis of MS.
2	15% blasts, No basophilia	70% blasts (CML- Blast crises)	t(9;22)	Ponatinib + FLAG-Ida therapy	Relapsed with blast crises, and death 2 months afterward
3	33% blasts + promyelocytes	28% promyelocytes (AML-M3, hyper granular variant)	Del(16q)	ATRA therapy	Death within a month of diagnosis of MS
4	15% blasts	24% blasts + promyelocytes (Secondary AML)	Monosomy 7	Decitabine therapy	Death after 09 months of diagnosis of MS

## DISCUSSION

This case series presents relatively rare cases of myeloid sarcoma by way of their site of involvement, presentation as well as the associated cytogenetic abnormality. Myeloid sarcoma is also known as Chloroma, Granulocytic Sarcoma, Myeloblastoma, and Extramedullary Leukemia. The term “Chloroma” was devised historically because of its greenish color (Chloros = Green in Greek), which is because of its high MPO content. Its association with acute leukemia was reported by Dock and Warthin in 1904.^2^ Burns^3^ reported the initial case of myeloid sarcoma (MS) and labeled it chloroma in 1823, while the first case of MS associated with AML was described in 1903 by Turk,^3^ who proposed the same basis for both the tumors. Rappaport^4^ introduced the term ‘granulocytic sarcoma’ in 1966.

The exact mechanism of occurrence of MS is unclear. However, extramedullary infiltration firmly denotes the presence of another homing signal that allows the blast cells to re-localize to secondary sites.^5^ In patients of AML with MS, an important element accountable for the migration of AML cells into the non-myeloid regions is the interaction between the matrix metalloproteinase (MMP) – 9 and leukocyte β2 integrin along with some unidentified protein complexes, known as ‘invadosomes’.^6^

MS occurrence has a mild predisposition for males (M:F = 2:1), with 60% of patients younger than 15 years of age.^6^ However, it is known to show a varied age distribution ranging from 1 year to 81 years of age.^7^ The most common anatomic sites of involvement are the skin (26%), lymph node (15%), testis (6%), and intestine (6%). Uncommonly, it may involve the bone (3%), CNS (3%), and biliary tract (3%). Other sites in the body are rarely involved.^8^

It typically presents as a part of AML (case1&3), or, less commonly, with a myeloproliferative neoplasm (case 2) or myelodysplastic syndrome (case 4).

MS is most commonly associated with AML. It has an incidence of 2-8% in these patients and can occur either as a unifocal or a multifocal neoplasm. It has been known to herald AML by months or years in approximately 25% of cases, appear collaterally with AML in 15% to 35% of cases, or infest after diagnosis in up to 50% of cases. It can also appear as a primary manifestation of relapse in a previously treated AML patient in remission.^7^ Additionally, in recent years, there have also been increasing reports of the development of MS following allogeneic stem cell transplantation, manifesting either as an isolated disease or along with relapse within the bone marrow.^8^^-^12 A crucial event in the pathogenesis may be the development of a novel chimeric gene and message as the result of the fusion of two genes: ETO from chromosome 8 and AML1 from chromosome 21.^13^

MS is comparatively more common in patients with AML with a predominant monocytic differentiation, namely myelomonocytic (French-American-British-FAB classification – M4) and monocytic (AML-M5) leukemia. However, according to Çakan et al.,^14^ MS may be associated with AML M2 more frequently than other subgroups. Our patient was a diagnosed case of AML-M2.

The prevalence of MS in patients with AML with translocation t(8;21) in various studies ranges between 9% and 35%.^15^^,^^16^ The frequent site of involvement in these cases was found to be the orbit. Breast involvement, seen in our patient, is relatively uncommon, and usually occurs in MS-associated with AML with inv(16). A historical retrospective study in patients with AML from Hiroshima and Nagasaki has reported breast involvement in approximately 8% of cases.^17^ Additionally, Pileri et al.,^8^ in their study in 2007, reported breast involvement in 2% of the cases. Other genetic deviations noted in such cases include t(15:17), t(9:11), t(1:11), t(8:17), del(16q), del(5q), del(20q), monosomy 7, trisomy 4 and trisomy 8.^18^

MS-associated with APML and CML is very rare. In CML, MS can occur mainly in the backdrop of blast phase (BP) or accelerated phase (AP). According to the 2016 World Health Organization criteria, CML-BP can be diagnosed when blasts are ⩾ 20% in the BM or peripheral blood (medullary BP); or there is an extramedullary blast proliferation, in other words, MS.^10^ In most CML-BP, the blast lineage is myeloid, and may include neutrophilic, monocytic, megakaryocytic, basophilic, eosinophilic or erythroid blasts, or any combination thereof. Flowcytometry is the preferred technique for phenotypic analysis of CML blasts in order to detect mixed phenotypes, but immunohistochemical stains can also be applied if a marrow aspirate cannot be obtained and there are insufficient numbers of blasts in the blood.^10^

The exact incidence of MS occurring in CML has not been mentioned in the literature. However, Chen et al.,^19^ in their study of 307 patients diagnosed with CML, reported 42 cases with MS. These had a male predilection with a male-to-female ratio of 4.3:1. The median age was 49.2 years at diagnosis of MS and ranged from 19.4 to 82.7 years.

Additionally, very few cases of MS in CML have been published in the literature and include isolated case reports of the occurrence of MS in CML at unusual clinical sites. Some of them have mentioned lymph node involvement. However, very few of them had MS as a presenting feature leading to the diagnosis of CML. In a report published by Bangerter et al.,^20^ out of the 26 patients, MS was the presenting feature of CML in only 01 patient. Similarly, Paydas et al.,^21^ studied 32 cases of MS. It was associated with AML in 13 cases, ALL with the presence of myeloid blasts in 01 case, CML in 11 cases, and MDS in 02 cases. MS was simultaneously diagnosed with leukemia in 05 cases and preceded leukemia in 08 cases. Lymph node and soft tissue were the frequently involved sites. Also, among these, 07 cases had been initially misdiagnosed as NHL, which had also been the scenario in our case.

Table 3 mentions the cases of CML reported in literature where MS was the 1st presentation. Cases not reported in English language and below 10 years of age have been excluded.^28^

**Table 3:** Previous reported cases of CML with initial presentation as MS

Authors	Age y/sex	Clinical feature	diagnosis	outcome
Torres et al.^22^	18/F	Iliac granulocytic sarcoma, Relapse as CML (blast crisis), Second relapse CML-BC, with CNS involvement, Progression of iliac tumor	diagnosed as CML (CP), CML (blast crisis), CML (blast crisis)	Died after the fourth presentation
Nagarajarao et al.^23^	53/M	Multiple skin nodules on the right side of his chest, right arm, left thigh and left shin	CML (CP)	Survived
Chen et al.^24^	14/M	Right inguinal lymphadenopathy (3 months)	CML presenting as mixed phenotype (T/ myeloid, bilineal) blast phase	Survived, in remission
Kumar et al.^25^	25/F	Localized swellings over posterolateral aspects of both thighs (2 months)	CML (chronic phase) presenting as MS	Survived
Levy et al.^26^	35/M	Right posterior shoulder pain	CML (Blast crisis) presenting as MS	Survived
Ai et al.^27^	23/F	Right inguinal lymphadenopathy (7 months)	CML (Blast crisis) presenting as MS (e1a2 BCR-ABL1 transcript)	Survived
Dhar et al.^28^	45/F	Swelling in the neck	CML (Blast crisis) presenting as MS	In remission.
Vassallo et al.^29^	50/M	Hoarseness of voice (laryngeal mass) with progressively growing left cervical lymph node mass along with dyspnoea	CML (Accelerated phase) presenting as MS	Died during therapy
Our case	73/M	Swelling in the neck	CML (Blast crisis) presenting as MS	Died after relapse

In patients with APML, MS commonly occurs in the relapse phase. In very rare cases, it may precede or may coincide with APML, as seen in our case. The most common sites of involvement are the skin and central nervous system (CNS). High white blood cell count (WBC) and younger age are suggested as risk factors.^22^

Additionally, increased incidences of MS have been noted since the introduction of ATRA. The literature mentions two possible theories for this increasing trend. One theory suggests a direct effect of ATRA on adhesion molecules, resulting in the increased infiltrative capability of leukemic cells. The second theory cites extended survival of the leukemia cells induced by gene‐targeting ATRA and ATO therapies.^23^ Our case developed MS during ATRA therapy.

It is also to be noted that this patient had isolated del(16q) as the genetic abnormality in APML (PML-RARA negative APML). Isolated deletion of the long arm of chromosome 16 [del(16q)] is rare in myeloid neoplasms with very limited data available in the literature, and is often associated with a complex karyotype. Historically, it was considered to be a variant of inv(16). However, studies have proved that it is distinct from the latter. Del(16q) is associated with myeloid neoplasms of the elderly (most commonly occurring in 6th decade), shows a heterogenous morphology and a relatively poor clinical outcome. It is associated with shorter overall survival and low rates of complete remission following chemotherapy.^24^

The exact pathogenesis of del(16q) in myeloid neoplasms is still unclear. Studies have suggested derangement or loss of genes located on 16q to play a role in its pathogenesis.^25^ Only 20 cases of myeloid neoplasms with del(16q) have been reported in the literature so far.^26^^,^^27^^,^^30^ Our case of pediatric APML with del(16q) presenting with MS during ATRA therapy, therefore, makes for a relatively unique and rare case.

Similarly, very few cases of MS in MDS have been described. MDS Patients with high-risk of developing MS are characterized by an elevated blast count with or without poor prognostic cytogenetics, which is considered to represent a leukemic transformation. Leukemic transformation in MDS occurs in about 15-17% of patients after 5 to 6 years of diagnosis.^31^ MS in MDS with monosomy 7 is quite infrequent. In the literature review done by Showalter et al.,^31^ of the 23 cases of MS in MDS reported in the literature, only 01 cases had monosomy 7.

Also, MS in the female genital tract occurs more commonly in the ovary. Few cases have been reported to arise in the uterus, vulva, or vagina. The literature mentions very few cases of MS involving the cervix.^32^^,^^33^ Moreover, most of these cases are associated with AML. Our case of MDS-EB2 presenting with MS as a presenting feature of relapse with AML transformation is very uncommon. Table 4 gives a summary of cases of MS in MDS reported in the literature.^31^

**Table 4:** Previous reported cases of MS in MDS

Authors	Age y/sex	Location	MDS type
Ravandi-Kashani et al.^34^	26/M	Mediastinum	MDS-EB2
Geisse et al.^35^	60/M	Tonsils	MDS-RS
Paydas et al.^21^	52/M	Lymph nodes	Hypoplastic MDS
Paydas et al.^21^	26/M	Lymph nodes	MDS-RS
Pileri et al.^8^	NA	Lymph Node	MDS with isolated del 5q
Pileri et al.^8^	NA	Skin	MDS with 5q- and monosomy 7
Cheng et al.^36^	57/M	Tonsil	MDS unclassifiable
Cornfield^37^	79/M	Spinal cord	MDS with complex cytogenetics
Grantham et al.^38^	56/M	Bladder	MDS- EB2
Koppisetty et al.^39^	66/M	Prostate	MDS-EB1
Showalter et al.^31^	77/F	Spinal cord	MDS with isolated del 5q
Our case.	49/F	Uterine cervix	MDS-EB2 with monosomy 7

Immunohistochemistry shows CD68-KP1 as the most commonly expressed marker. However, CD117, MPO, CD43, CD34, lysozyme, CD56, and CD99 are the commonly used markers for the diagnosis of MS. Among these, CD43 and lysozyme are the most sensitive markers. The genetic abnormalities associated with MS include MLL gene rearrangement, t(8;21), t(15:17), t(9:11), t(1:11), t(8:17), del(16q), del(5q), del(20q), monosomy 7, trisomy 4 and trisomy 8. t(8:21) has been reported as the most common cytogenetic aberration associated with MS, occurring both at presentation and upon relapse, and typically involves the orbit in infants.

Management modalities of MS include systemic and local treatment involving chemoradiotherapy, targeted therapy, and bone marrow transplantation.

The outcome of patients suffering from MS is usually poor. Literature mentions an approximate 5-year survival rate of 20% with appropriate therapeutic interventions.^40^ Most patients succumb within a year of diagnosis of MS. The most frequent causes of death are infections and relapse.^40^ Among the prognostic factors, a relatively poorer prognosis is associated with the presence of FLT3 mutations, MS with t(8;21),^41^ abnormalities of chromosome 89, development of MS in age ≤15 years, development of MS in CML and/or MDS,^40^ and involvement of CNS, soft tissue or lymph node.^42^ MS is also known to show a racial disparity: blacks have a poorer outcome than whites.^42^ Additionally, a study conducted by Goyal et al.,^42^ showed that early systemic chemotherapy among older patients (age ≥ 70 years) with MS was associated with the worst prognostic outcome, as compared to early radiotherapy or surgical resection or no therapeutic intervention at all. In this case series, all of the patients presented with one or more of the above-mentioned poor prognostic factors, leading to their early deaths. Longer follow-up periods with an early diagnosis and therapeutic intervention may help in improving the survival of such patients.

## CONCLUSION

MS is an uncommon neoplasm associated with myelogenous neoplasms/leukemias. It can present at any site of the body and at any time during the disease course. This case series depicts a varied presentation of MS. Due to the versatility of its clinical presentation, it is important to consider a differential diagnosis of MS in hematologic neoplasms, small round cell undifferentiated neoplasms and undifferentiated carcinomas/sarcomas, especially when CD20 and CD3 IHCs are non-reactive, even in the absence of clinically evident leukemia or MDS. Simultaneous hematological work up with a hemogram and a peripheral blood smear further necessitates the accurate histological diagnosis. Timely diagnosis of MS goes a long way in the management of the disease, thereby improving the survival of the patient.
